# Synergistic Enhancement of Protein Recruitment and Retention via Implant Surface Microtopography and Superhydrophilicity in a Computational Fluid Dynamics Model

**DOI:** 10.3390/ijms242115618

**Published:** 2023-10-26

**Authors:** Hiroaki Kitajima, Makoto Hirota, Toshinori Iwai, Kenji Mitsudo, Juri Saruta, Takahiro Ogawa

**Affiliations:** 1 Weintraub Center for Reconstructive Biotechnology, UCLA School of Dentistry, Los Angeles, CA 90095-1668, USA; hiroaki_k_0315@yahoo.co.jp (H.K.); mhirota@yokohama-cu.ac.jp (M.H.); saruta@kdu.ac.jp (J.S.); 2 Division of Regenerative and Reconstructive Sciences, UCLA School of Dentistry, Los Angeles, CA 90095-1668, USA; 3 Department of Oral and Maxillofacial Surgery, Graduate School of Medicine, Yokohama City University, 3-9 Fukuura, Kanazawa-ku, Yokohama 236-0004, Kanagawa, Japan; iwai104@yokohama-cu.ac.jp (T.I.); mitsudo@yokohama-cu.ac.jp (K.M.); 4 Department of Oral and Maxillofacial Surgery/Orthodontics, Yokohama City University Medical Center, 4-57 Urafune-cho, Minami-ku, Yokohama 232-0024, Kanagawa, Japan; 5 Department of Education Planning, School of Dentistry, Kanagawa Dental University, 82 Inaoka, Yokosuka 238-8580, Kanagawa, Japan

**Keywords:** bone-implant integration, microrough, osseointegration, titanium dental and orthopedic implant, UV photofunctionalization

## Abstract

The exact mechanisms by which implant surface properties govern osseointegration are incompletely understood. To gain insights into this process, we examined alterations in protein and blood recruitment around screw implants with different surface topographies and wettability using a computational fluid dynamics (CFD) model. Compared with a smooth surface, a microrough implant surface reduced protein infiltration from the outer zone to the implant thread and interface zones by over two-fold. However, the microrough implant surface slowed blood flow in the interface zone by four-fold. As a result, compared with the smooth surface, the microrough surface doubled the protein recruitment/retention index, defined as the mass of proteins present in the area per unit time. Converting implant surfaces from hydrophobic to superhydrophilic increased the mass of protein infiltration 2–3 times and slowed down blood flow by up to two-fold in the implant vicinity for both smooth and microrough surfaces. The protein recruitment/retention index was highest at the implant interface when the implant surface was superhydrophilic and microrough. Thus, this study demonstrates distinct control of the mass and speed of protein and blood flow through implant surface topography, wettability, and their combination, significantly altering the efficiency of protein recruitment. Although microrough surfaces showed both positive and negative impacts on protein recruitment over smooth surfaces, superhydrophilicity was consistently positive regardless of surface topography.

## 1. Introduction

Endosseous titanium implants with microtopographic surfaces have become standard in dental and orthopedic practice [1,2,3,4,5,6,7,8,9,10,11]. Commonly used microrough surfaces are created via acid etching and present compartmental structures consisting of sharp peaks and valleys of 0.5–3 μm height and width [12,13]. Microtopography not only strengthens the mechanical interlocking between the bone and implant surface but also promotes the differentiation of osteogenic cells compared with relatively smooth machined surfaces [14,15,16,17,18,19,20,21,22,23,24,25,26,27,28,29,30,31,32], thereby accelerating bone formation around implants [33,34]. However, there remain unanswered questions around the eventual bone phenotypes that develop around implants with microtopographic surfaces. For instance, less bone develops around microrough implants than around machine-smooth implants. Although this might be because osteoblast proliferation is slower on microrough surfaces, other mechanisms might contribute. Bone forms close to microrough surfaces (contact osteogenesis), whereas bone formation around smooth surfaces is relatively distant (distant osteogenesis) [35,36,37]. It has been shown that unique molecular layers form exclusively at the microrough titanium interface, which enhances the mechanical and adhesive properties of the interfacial tissue [38]. Rapid bone formation is seen around microrough implants with minimal soft tissue intervention. While these distinct osteogenic effects may be due to faster osteoblast differentiation on microrough surfaces, this might not be the only explanation and, more importantly, the reason for the faster osteoblastic differentiation has not been fully established. In particular, there are few data on the role of protein and blood localization on and around implants, even though this is crucial for cellular recruitment, attachment, and signaling in peri-implant osteogenesis. Specifically, it is unknown how proteins are recruited to implant surfaces via blood flow and whether this is influenced by surface topography.

The recent implementation of computational fluid dynamics (CFD) in implant science has provided a new in silico approach to understanding osseointegration by simulating blood and protein flow around implant surfaces [39,40]. In these studies, CFD models mimicking standard-sized, screw-shaped dental implants were created and located in bone. To simulate a clinical scenario, the implant was surrounded by bone with a spacious gap sufficient to let blood flow. The blood inlet was bidirectional from the apex of the implant and surrounding bone. The studies demonstrated that the CFD model is simple, low-cost, and useful to explore the new area of research in implant biology and, indeed, provided novel results to deepen the understanding of osseointegration. For instance, macroscopic implant morphology, such as the screw shape or implant threads, induces protein retention by slowing down blood flow in certain areas of the implant [41]. Compared with hydrophobic surfaces, superhydrophilic implant surfaces—where the contact angle of water θ is 0°—effectively promote the recruitment of proteins to the implant interface [39,40]. These results provided new insights into the regulation of protein and cell recruitment to implant surfaces, which is key to successful osseointegration. Furthermore, protein and blood localization is not accidental nor solely dependent on their interaction with the implant surface but also on their dynamic flow on a larger scale, including the remote zone outside the implant threads. These facets of fluid dynamics cannot be addressed in cell culture studies and are extremely difficult to model in vivo.

Surface topography and physicochemistry are major factors determining the biological capability of titanium implants [42,43,44,45,46,47,48]. Specifically, hydrophilicity/hydrophobicity, or wettability, is a major physicochemical property [43,44,45,46]. Although ordinary titanium surfaces, regardless of surface topography, are hydrophobic [49], hydrophilic surfaces can appear under certain conditions and be induced by some surface modifications [50,51,52]. In particular, ultraviolet (UV) light treatment induces the superhydrophilicity of titanium surfaces, providing a new means to improve osseointegration both experimentally [53,54,55,56,57,58,59,60,61,62,63] and clinically [45,53,64,65].

Therefore, the objective of this study was to use CFD to model and compare protein and blood dynamics around screw-shaped implants with amorphous, smooth surfaces or microrough surfaces, in particular focusing on the mass and speed of infiltration of blood and protein in the vicinity of implant surfaces. The combinational effects of surface topography and hydrophilic/hydrophobic state were also examined. Fibrinogen was used as a model protein because it is critical to bone wound healing.

## 2. Results

### 2.1. Visualizing Fibrinogen through Color Mapping

**Figure 1:**
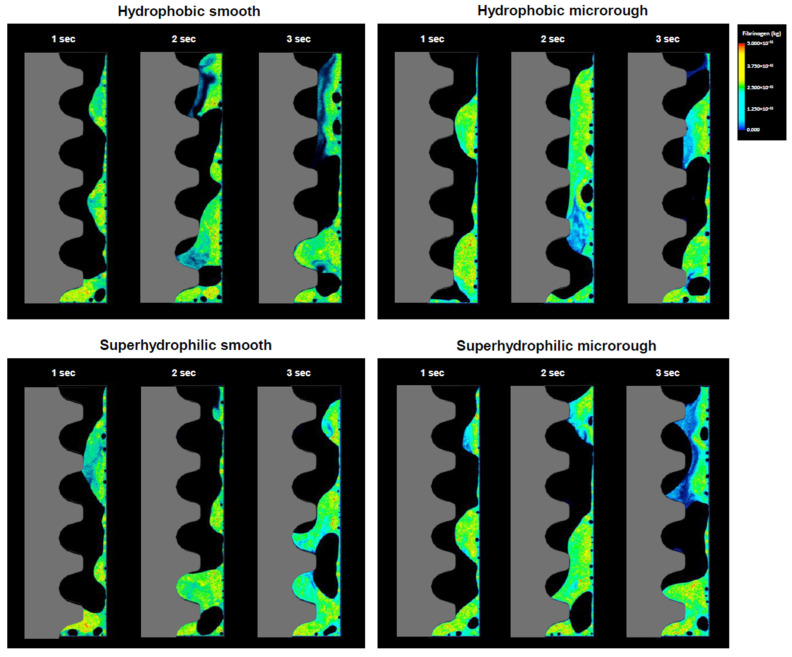
Fibrinogen dynamics visualized via color mapping. Implants with two different surface topographies and with or without superhydrophilicity are compared. The color scale applies to all panels.

We first visualized the localization of fibrinogen along the implant surface by drawing density-based color maps during blood flow. Blood flowed from the implant apex and surrounding bone. After 1 s, the first apical thread but not the other threads of the hydrophobic (contact angle 70°) smooth implant was filled with fibrinogen but, by 3 s, fibrinogen had infiltrated into the second implant thread (Figure 1). Around hydrophobic microrough implants, even the first apical thread was only half-filled with fibrinogen, and there was no progressive infiltration into the other threads.

Fibrinogen filled the first, second, and third threads of the superhydrophilic (contact angle 0°), smooth implant at 1, 2, and 3 s, respectively. The superhydrophilic microrough implant also showed progressive infiltration, with the 3 s map showing fibrinogen even in the fourth and fifth threads, significantly different from the hydrophobic microrough implant.

### 2.2. Longitudinal Fibrinogen Quantification

**Figure 2:**
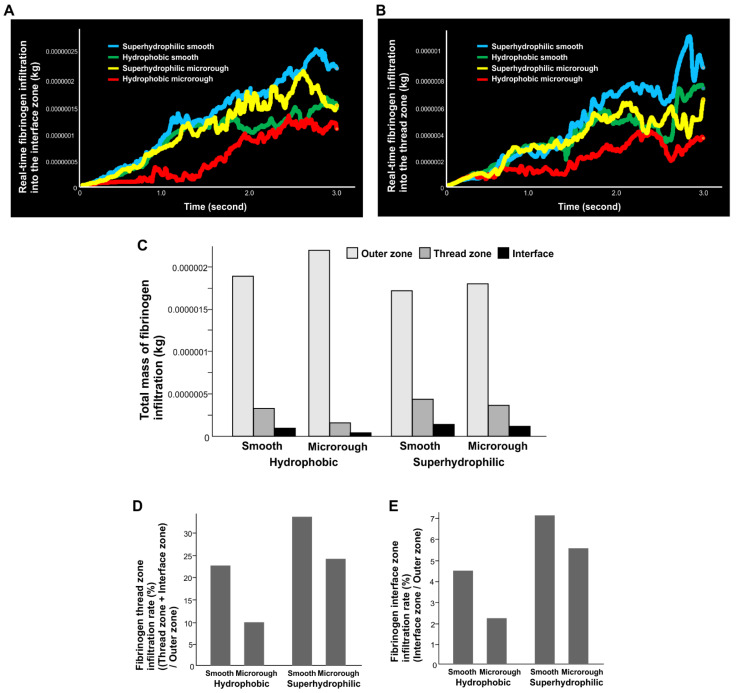
Quantitative assessment of time-dependent fibrinogen quantity infiltrating into each of the interface (**A**) and thread (**B**) zones. (**C**) Total mass of fibrinogen present in each of the three different zones. (**D**) The ratio of fibrinogen distribution in the thread zone relative to the one in the outer zone. (**E**) The ratio of fibrinogen distribution in the interface zone relative to the one in the outer zone.

We next quantified the mass of fibrinogen infiltrating each of the interface and thread zone areas relevant to osseointegration (Figure 2). In the interface zone of the hydrophobic smooth surface (Figure 2A), the mass of fibrinogen increased nearly linearly over time, whereas infiltration was delayed around the hydrophobic microrough surface. Making the surface superhydrophilic (contact angle 0°) considerably increased the fibrinogen infiltration of both smooth and microrough surfaces in the order of superhydrophilic smooth, superhydrophilic microrough, hydrophobic smooth, and hydrophobic microrough surfaces.

There was also greater infiltration into the thread zone of the hydrophobic smooth surface than the hydrophobic microrough surface (Figure 2B). The fibrinogen mass around the hydrophobic microrough interface was only 50% of that around the counterpart smooth implants, even after 3 s. Superhydrophilicity significantly increased fibrinogen infiltration for both surface topographies. Similar to the result of the interface zone, the most fibrinogen was around the superhydrophilic smooth surface, and the least was around the hydrophobic microrough surface.

### 2.3. Fibrinogen Distribution among Three Zones

We next quantified the total mass of fibrinogen localizing to the interface, thread, and outer zones (Figure 2C). The results confirmed that there was more fibrinogen infiltration (1) into the interface and thread zones of the smooth surface than the microrough surface, and (2) for the superhydrophilic surfaces than for the hydrophobic surfaces. Of note, there was more fibrinogen infiltration into the interface and thread zones of the superhydrophilic microrough surface than the hydrophobic smooth surface, indicating that superhydrophilicity could even overcome the negative effect of the microrough surface. Accordingly, fibrinogen localization in the outer zone was greater for microrough and hydrophobic surfaces due to less influx into the thread and interface zones.

To evaluate the volumetric rate of protein recruitment, particularly to areas relevant to osseointegration, we next calculated the percentage of fibrinogen in the interface and thread zones relative to that in the outer zone (Figure 2D). Although protein recruitment rates at microrough surfaces were lower, superhydrophilic conversion of the surfaces significantly improved it, i.e., the effect of superhydrophilicity was greater for microrough surfaces (Figure 2D,E). The fibrinogen infiltration rate was even higher in the interface zone than the thread zone for superhydrophilic surfaces, resulting in a higher infiltration rate for the superhydrophilic microrough surface than for the hydrophobic smooth surface (Figure 2E).

### 2.4. Vector Mapping

**Figure 3:**
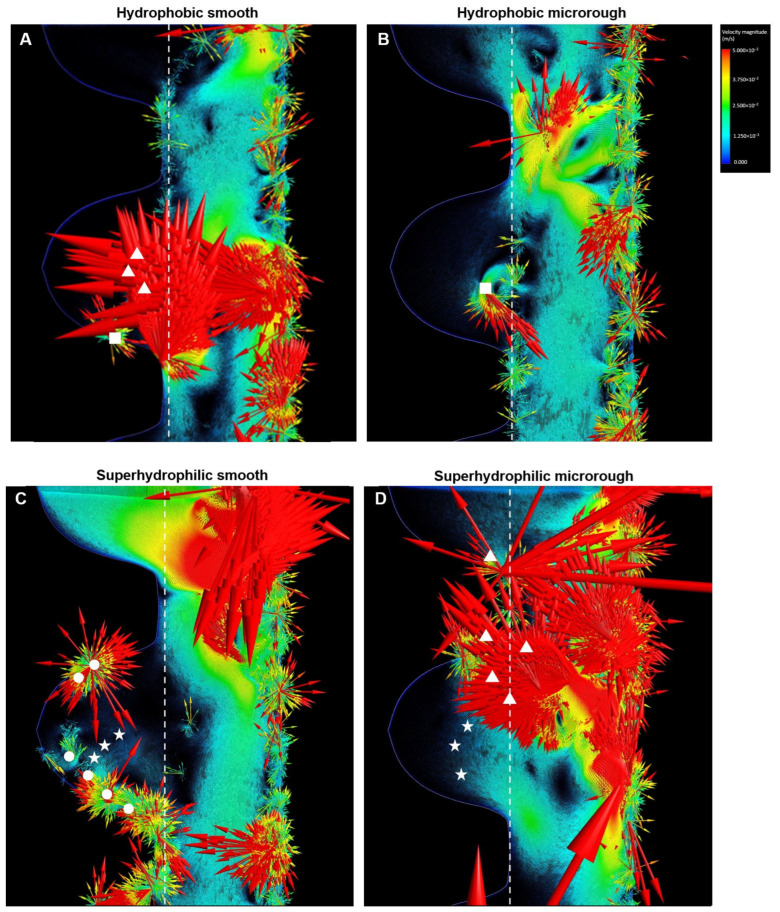
Vector color mapping for blood flow around four different surfaces (**A**–**D**). Each vector represents the direction and speed of the cell meshed in the domain. Refer to the main text for symbols. The color scale applies to all panels.

We next created a vector field formation map for whole blood, and representative vector maps after 2 s of blood inflow are shown in Figure 3. There was robust inbound vector formation across the thread and outer zone border (white dotted lines) for the smooth hydrophobic implant (white triangles in Figure 3A), with no current within the implant threads except for small multidirectional vector clusters indicative of vortex initiation (white squares in Figure 3A). Similarly, the hydrophobic microrough implant did not show structured vector formation within the thread except for minor vortex formation (white squares in Figure 3B). Unlike the smooth implant, there was no solid inbound vector at the entrance of the thread zone around the microrough implant.

Fibrinogen reached the interface zone around the superhydrophilic smooth implant, showing arrays of dense vector formation along the implant interface (white circles in Figure 3C) and current formation inside the thread (white stars in Figure 3C). The superhydrophilic microrough implant showed rigorous formation of inbound, long vectors at the outer thread zone border (white triangles in Figure 3D) and current inside the thread (white stars in Figure 3D).

### 2.5. Blood Velocity

**Figure 4:**
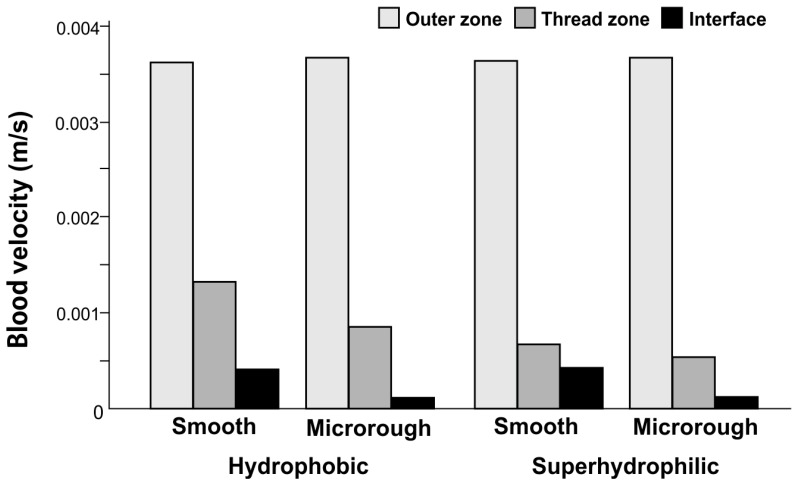
Average velocity of blood flow calculated from the vector analysis.

We next analyzed the average velocity of the whole blood based on the vector analysis. Velocity was highest in the outer zone for all four implant surfaces, followed by the thread and interface zones (Figure 4). Although the outer zone velocity was similar for the four surfaces, the thread and interface zone velocities were substantially different according to surface type. Thread–zone velocity was higher for smooth surfaces than microrough surfaces, and superhydrophilicity significantly reduced the velocity. The interface velocity was also higher for smooth surfaces than microrough surfaces, but the effect of superhydrophilicity/hydrophobicity was not substantial. The velocity varied drastically according to surface topography, wettability, and zones, for example: (1) the thread zone velocity for the superhydrophilic microrough surface was half that of the hydrophobic smooth surface; (2) the interface velocity was 2.7-fold slower for the superhydrophilic microrough surface than the hydrophobic smooth surface; and (3) the interface velocity was 25-fold slower than the outer zone velocity around the superhydrophilic microrough surface.

### 2.6. Fibrinogen Recruitment/Retention Index

**Figure 5:**
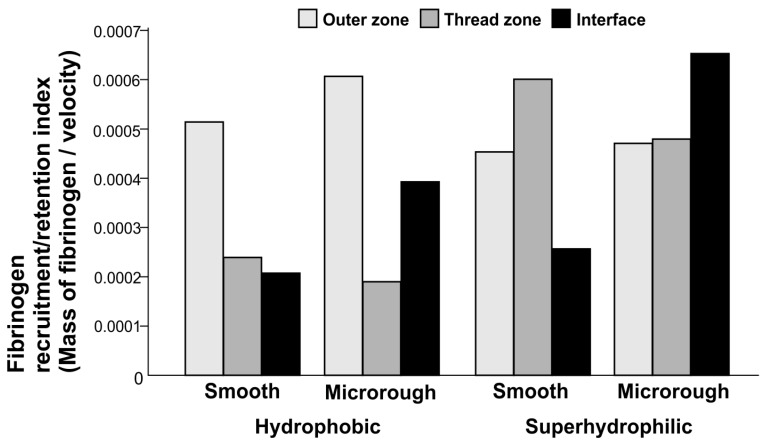
Fibrinogen recruitment/retention index (y-axis) defined as the mass of fibrinogen present in a particular zone per time unit and calculated according to the mass of fibrinogen infiltration in the zone divided by blood velocity.

We hypothesized that both circulating protein mass and protein retention are important in the local microenvironment for osseointegration. We, therefore, analyzed how fibrinogen localized around implants by calculating the fibrinogen recruitment/retention index, which was defined as the total mass of fibrinogen divided by the blood velocity (Figure 5). This index represents the quantity of fibrinogen localizing to an area of interest per unit time. The fibrinogen recruitment/retention index varied considerably with surface topography and in different zones. The index was highest in the outer zone, followed by the thread and interface zones, around the hydrophobic smooth surface. However, it was highest in the interface zone around the hydrophobic microrough surface, with an approximately 2-fold increase compared with the hydrophobic smooth surfaces.

Adding superhydrophilicity to smooth surfaces significantly increased the fibrinogen recruitment/retention index in the thread zone by 2.5-fold. Likewise, superhydrophilic microrough surfaces showed a significant increase in the thread and interface zones compared with hydrophobic counterparts. The index in the interface zone of the superhydrophilic microrough surface was the highest of all the tested zones and surface types.

## 3. Discussion

This is the first study to analyze the effects of two different implant surface properties—topography and wettability—on fibrinogen infiltration and speed of blood flow on and around titanium screw implants. We hypothesized that the biologically meaningful recruitment of proteins to the implant surface depends not only on the total mass of proteins delivered adjacent to an implant surface but also on protein retention in this area. According to this hypothesis, slow rather than fast movement of blood would be beneficial for localizing proteins adjacent to implant surfaces. By implementing a unique index representing both the recruitment/retention of fibrinogen (the higher the index, the greater the mass of fibrinogen in the area-per-unit time), we found that fibrinogen recruitment/retention significantly varied with implant surface topography and wettability and in different zones around the implant. Given that implant anchorage depends on bone formation primarily at the implant interface and secondarily in the thread zone, the index at the interface zone is of the most importance, followed by the thread zone. In the interface zone, microrough surfaces had higher indices than smooth surfaces, and adding superhydrophilicity to the microrough surfaces further increased this advantage. The index in the thread zone was less affected by surface topography but was considerably increased by superhydrophilicity for both smooth and microrough surfaces. Of note, the index, which was lowest in the interface zone and highest in the outer zone for the smooth implant, was completely opposite around superhydrophilic microrough implants, indicating a complete reversal in protein recruitment/retention through the synergistic effect of microtopography and superhydrophilicity.

**Figure 6:**
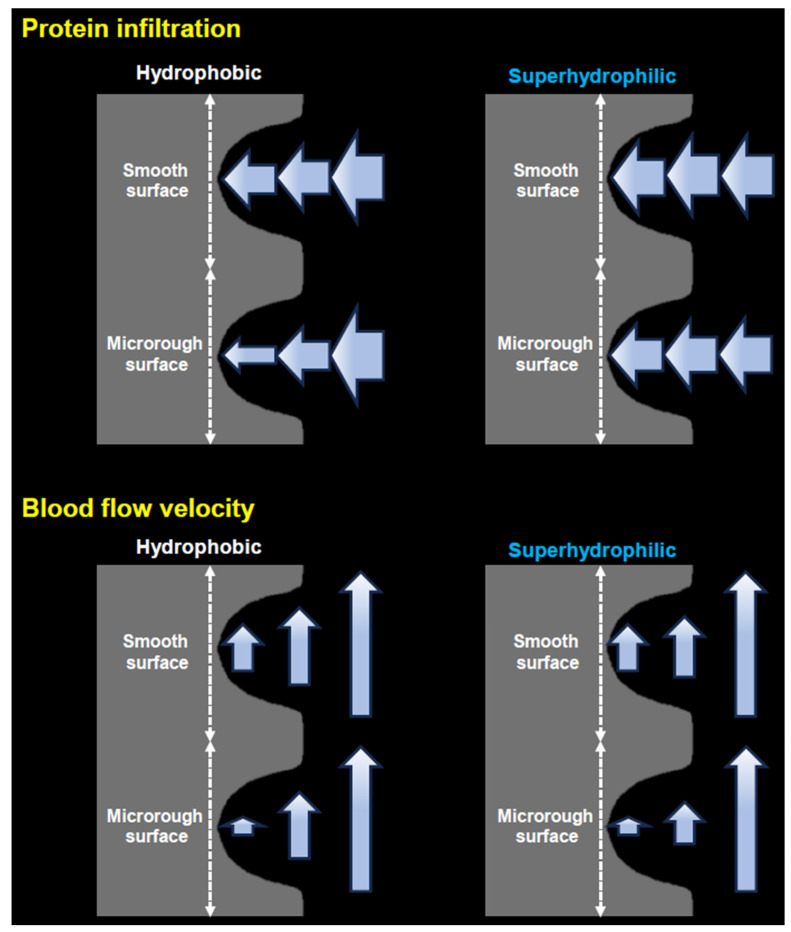
A diagram highlighting protein infiltration and blood speed regulated by surface topography and wettability. The widths of arrows represent the mass of protein infiltration in upper panels, while the lengths of arrows represent the speed of blood flow in bottom panels. The three arrows in each panel denote their location at the outer, thread, and interface zones.

This study revealed distinct control of the mass and speed of protein and blood flow through implant surface properties (see schematic, Figure 6). Interestingly, under hydrophobic conditions, the microtopography of implant surfaces significantly reduced the infiltration of fibrinogen from the outer zone to the thread and interface zones, which would be unfavorable for osseointegration, but it slowed down blood flow, which would be favorable for osseointegration. The mass of fibrinogen in the thread and interface zones of microrough surfaces was less than half of that at smooth surfaces. Instead, the blood speed at the interface zone around the microrough surface was significantly (four-fold) reduced compared with around the smooth surface, which attenuated the mass disadvantage and resulted in an even better recruitment/retention index.

By contrast, rendering implant surfaces hydrophilic only had a positive impact on both the mass and speed of proteins and blood flow. Superhydrophilic surfaces markedly increased fibrinogen influx to the thread and interface zones, regardless of the surface topography, with a notable increase for the microrough surface, mitigating its disadvantage over the smooth surface. Although superhydrophilicity did not significantly influence the blood flow speed in the interface zone, it slowed down the blood by over 50% in the thread zone. Thus, the fibrinogen recruitment/retention index was markedly increased in the thread zone of the superhydrophilic, microrough surface.

This study also revealed the effect of the macroscopic morphology of implants on protein and blood flow. The area of the thread zone was one-quarter that of the outer zone. Therefore, 25% of inlet fibrinogen should have been distributed into the thread zone. In reality, only 23% and 9% of fibrinogen infiltrated into the thread zone for the smooth and microrough surfaces, respectively, under hydrophobic conditions, suggesting that the screw-shaped configuration limits blood infiltration. However, blood speed was 60–70% lower in the thread zone than in the outer zone, producing a bay effect. Together, the macroscopic implant threads had both a positive and negative role on the recruitment of proteins and blood, providing a new perspective on the importance of implant screw morphology, including, but not limited to, the pitch, depth, and angle of threads, in addition to its main purpose of obtaining primary stability in surgery. When considering new designs, the wettability of the surfaces should also be taken into account.

Increased protein adsorption and cell retention on microrough implant surfaces have been considered underlying mechanisms promoting osseointegration compared to that occurring around machined, smooth implant surfaces [28,66,67], probably due to the increased surface area from the micro-configuration and increased mechanical interlocking of proteins and cells with the complex surface morphology, as supported by in vitro data. Although direct comparisons of in vitro data and in silico simulations should be interpreted with caution, the significantly reduced mass of protein recruitment and significantly slowed protein and blood flow around the microrough surfaces seen here provide novel insights into surface topography-enhanced osseointegration. Indeed, the protein recruitment/retention index in the interface zone of microrough surfaces was double that for smooth surfaces, which may explain the contact osteogenesis seen histologically around microrough surfaces [35,36].

This study had several limitations. First, in CFD analyses, there is no deviation or variation in results, i.e., the results will be identical under the same conditions. However, there could be potential errors arising from the number and quality of the computational mesh during modeling and calculation protocols like truncation errors. In this study, the mesh on each boundary consisted of the same length of edges for all experimental groups, with all analytical factors and conditions being consistent among the groups, establishing the reliability of the results. We also attempted to further increase the reliability by analyzing cumulative, quantitative data in addition to the cross-sectional, qualitative imaging and mapping. Second, the results should be interpreted within the scope of an in silico simulation, although the findings have value because blood and protein flow cannot be fully assessed in vivo using current technologies or approaches. We compared contact angles of 0° and 70° in our simulations, where a contact angle of 0° is expected on UV-treated titanium surfaces and when titanium surfaces are new [50]. The contact angle of 70° was specified because the contact angle of ordinary titanium specimens ranges from 65° to 110°, depending on the age and roughness of the surface, with rougher surfaces having a higher contact angle [50,68]. We used 70° for both smooth and microrough surfaces, but a higher contact angle could be considered for microrough surfaces to better mimic reality. Despite these limitations, the differences observed between different surfaces demonstrate the sensitivity and utility of implant CFD models for evaluating protein and blood dynamics. There are ongoing experimental and clinical efforts to develop new implant surfaces to improve osseointegration, and implant surfaces with nanotopography, meso-topography, and other morphological features [69,70,71,72,73] should now be tested using the CFD approach demonstrated here.

The mechanisms underlying increased osseointegration around UV-photofunctionalized implants are not fully understood but include the following: (1) enhanced cellular adhesion and proliferation via hydrophilic lipid ends on the cell membrane of osteoblasts reacting to superhydrophilic titanium surfaces; (2) enhanced cellular affinity facilitating the adhesion, spread, and proliferation on superhydrophilic titanium due to their hydrocarbon pellicle-free surfaces; and (3) increased attraction of cells to UV-treated titanium due to interactions between positively charged UV-treated titanium surfaces and negatively charged osteoblast cell membranes. The significantly increased recruitment/retention index around superhydrophilic surfaces due to the dual effects of the increased mass of protein infiltration and reduced speed of blood flow now provide another putative explanation. Although not examined here, the slow blood flow induced by microrough surfaces and combined microtopography and superhydrophilicity should also benefit the recruitment of circulating stem and osteogenic cells.

## 4. Materials and Methods

### 4.1. Computational Fluid Dynamics (CFD) Implant Model

**Figure 7:**
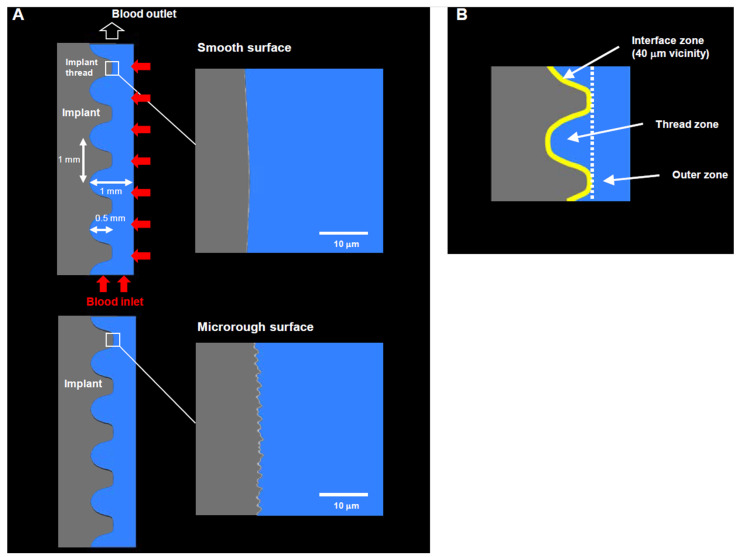
A CFD model designed for a screw-shaped implant. (**A**) Dimensions and design of an implant, blood area, boundary conditions, and modeling of two different surface topographies. (**B**) The three different zones defined around an implant.

A geometric model for implants was created using ANSYS Design Modeler (2019 R1, ANSYS Inc., Canonsburg, PA, USA) to simulate the environment around the implants, similar to previously [39,40] (Figure 7A). Two different topographies were modeled: (1) a smooth, amorphous surface with no projections or irregularities, mimicking the machined implant surfaces used in the field; and (2) a microrough surface with 0.5–3.0 μm random peaks and valleys mimicking a common microrough surface made via acid etching. The model, boundaries, blood inlet and outlet, and three analysis zones (interface, thread, and outer zone) were designed following methods reported elsewhere (Figure 7B) [40].

### 4.2. Numerical Conditions

We used an established analytical approach that included the volumes of fraction (VOF) model, species transport model, fluid properties, and numerical conditions [40]. Briefly, the VOF and species transport models in ANSYS Fluent (2019 R1, ANSYS Inc.) were utilized to analyze the flow of blood plasma, red blood cells (RBCs), fibrinogen, and whole blood. All equations used in the analysis are defined and described in the ANSYS Theory Guide and User’s Guide [74,75]. It was assumed that no chemical reaction happens between species, and the transfer of temperature was considered negligible. The boundary condition in this study assumed blood flow from capillaries distributed in alveolar bone. Studies that have measured RBC velocity in the capillaries have shown that their values range from 1.0 to 4.0 mm/s [76]. Therefore, the velocity at the blood inlet and in alveolar bone was set to 0.001 m/s. For the outflow boundary condition, a free stream boundary condition was used. The contact angle between blood plasma and the implant surface was set to 0° for a superhydrophilic surface or 70° a hydrophobic surface. A normal adult human hematocrit (45%) was used as the VOF value at the blood flow inlet and alveolar bone, so the VOF for blood plasma was 55%. The mass fraction of fibrinogen ($Y_{0}$) at the blood flow inlet and alveolar bone was 0.29% and was obtained by dividing the normal adult human fibrinogen concentration (300 mg/dL = 3 kg/m^3^) by the density of blood serum (1024 kg/m^3^) [77]. Time step size and the number of steps were set to 0.0001 s and 30,000, respectively. The pressure-based solver in ANSYS Fluent was used, as it was necessary for VOF modeling. The calculation at each time step was considered to have reached convergence when the rate of change in the mass flow of fibrinogen (kg/s) was below 0.001. A double-precision solver was used. Pressure–velocity coupling was achieved using the coupled scheme. Since Reynold’s number was sufficiently lower than the value at which the flow field transitions into a turbulent flow (i.e., 2800), the flow field within the fluid zone was considered laminar.

## 5. Conclusions

Using an implant CFD model, here, we revealed distinct control of the mass and speed of protein and blood flow via implant surface topography and wettability. The microrough implant surface reduced protein infiltration from the outer zone to the implant thread and interface zones compared with smooth surfaces. However, the microrough implant surface effectively slowed blood flow in the thread and interface zones. As a result, the protein recruitment/retention index of microrough surfaces was twice that for smooth surfaces. Converting implant surfaces from hydrophobic to superhydrophilic significantly improved protein and blood dynamics. Superhydrophilicity increased the mass of protein infiltration 2–3-times and further slowed the blood flow by up to two-fold in the implant vicinity for both smooth and microrough surfaces. Consequently, the protein recruitment/retention index was highest at the implant interface when the implant surface was superhydrophilic and microrough. CFD now provides scope to test many different combinations and types of surface topography and physicochemical properties to accelerate the development of implants that optimally promote osseointegration.

## Data Availability

Data availability on request from author.
